# Water Organic Pollution and Eutrophication Influence Soil Microbial Processes, Increasing Soil Respiration of Estuarine Wetlands: Site Study in Jiuduansha Wetland

**DOI:** 10.1371/journal.pone.0126951

**Published:** 2015-05-18

**Authors:** Yue Zhang, Lei Wang, Yu Hu, Xuefei Xi, Yushu Tang, Jinhai Chen, Xiaohua Fu, Ying Sun

**Affiliations:** 1 Key Laboratory of Yangtze River Water Environment, Ministry of Education, School of Environmental Science and Engineering, Tongji University, Shanghai, China; 2 Shanghai Jiuduansha wetland Nature Reserve Administration, Shanghai, China; University of Oklahoma, UNITED STATES

## Abstract

Undisturbed natural wetlands are important carbon sinks due to their low soil respiration. When compared with inland alpine wetlands, estuarine wetlands in densely populated areas are subjected to great pressure associated with environmental pollution. However, the effects of water pollution and eutrophication on soil respiration of estuarine and their mechanism have still not been thoroughly investigated. In this study, two representative zones of a tidal wetland located in the upstream and downstream were investigated to determine the effects of water organic pollution and eutrophication on soil respiration of estuarine wetlands and its mechanism. The results showed that eutrophication, which is a result of there being an excess of nutrients including nitrogen and phosphorus, and organic pollutants in the water near Shang shoal located upstream were higher than in downstream Xia shoal. Due to the absorption and interception function of shoals, there to be more nitrogen, phosphorus and organic matter in Shang shoal soil than in Xia shoal. Abundant nitrogen, phosphorus and organic carbon input to soil of Shang shoal promoted reproduction and growth of some highly heterotrophic metabolic microorganisms such as β-Proteobacteria, γ-Proteobacteria and Acidobacteria which is not conducive to carbon sequestration. These results imply that the performance of pollutant interception and purification function of estuarine wetlands may weaken their carbon sequestration function to some extent.

## Introduction

Soil is an important carbon pool that has stored large amounts of organic carbon while releasing CO_2_ via soil respiration (SR). Any increases in soil CO_2_ emissions in response to environmental change have the potential to exacerbate increasing atmospheric CO_2_ levels and provide positive feedback to global warming. As a result, the organic carbon sequestration ability of soil is of great importance to reducing greenhouse gas emissions and global warming. SR includes soil microbial respiration (SMR), root respiration, and soil animal respiration [1]. Soil respiration is obviously dependent on microbial activity and SMR [2], and many factors such as plant properties, soil structure and properties, soil utilization and fertilization affect microbial communities and soil microbial activity and therefore SR by changing the soil micro environment [3–5].

Undisturbed natural wetlands are important carbon sinks due to their low rate of organic decomposition and SR due to continually being flooded, which results in low temperature and hypoxia [6]. The incomplete decomposition of organic matter leads to carbon and nutrient accumulation in wetland soil and plants forming a huge carbon pool. As a result they have great potential for the exchange of greenhouse gases (CO_2_ and CH_4_) with the atmosphere, in which case, determination of whether there is a carbon sink or pool must been based on carbon budget. Most previous investigations of carbon sequestration of wetlands have been conducted in alpine inland wetlands [7–9]. These ecosystems store a large amount of soil organic carbon (SOC) due to their low decomposition rate [10]. The climate of the region is characterized by long cold winters, a short growing season, and cool summers with relatively high precipitation, which causes organic carbon added to the soil to be sequestrated for a long period of time [8]. The carbon sequestration capability of young estuarine salt marshes has been overlooked due to its short development history and thus the relatively low storage of SOC. However, its sequestration has attracted increasing attention over the past 20 years due to further global warming. Zhang et al. found that SOC and SMR varied significantly among different successional stages of tidal wetland in Chongming Dongtan [11]. Chmura et al. reported that, in contrast to peatlands, salt marshes and mangroves release negligible amounts of CH_4_ and sequester more carbon per unit area after compiling data for 154 sites in mangroves and salt marshes from the western and eastern Atlantic and Pacific coasts, as well as the Indian Ocean, Mediterranean Ocean, and Gulf of Mexico [12].

However, natural disturbances such as increasing sea level, typhoons and storm tides, climate change and anthropogenic disturbances such as reclamation, soil use, coastal engineering and environmental pollution may change soil structure and properties and the nature and activities of soil microorganisms, thus affecting the SR and carbon sequestration capability of wetlands [13]. Some scientists have found that anthropogenic and natural factors [14] such as agricultural drainage, land-use changes, increasing atmospheric CO_2_ concentrations and global climate change affected carbon sequestration of peatland, resulting in their shifting between carbon sinks and sources. Xi et al. found that a synergistic effect of increased temperature and sea level lead to an obvious acceleration in SMR and β-glucosidase activity [15].

When compared with inland wetlands, especially with alpine wetlands, estuarine areas are generally economically developed and densely populated, which means that land use and human disturbances including reclamation [16], hydraulic engineering construction [17] and pollution input [18] are more serious and threaten the preservation of estuarine wetlands and their ecological function [19]. Tang et al. found that siltation promotion and agricultural utilization led to changes in soil structure and characteristics of existing estuarine wetlands such as decreased water capacity and increased inorganic N, and that they may weaken wetland carbon storage capacity [20].

In addition to carbon sequestration ability, estuarine wetlands have the ability to purify and reduce pollution of surrounding water, such as organic matter and ammonia [21], which may change the soil microenvironment and consequently affect the soil microbial community, and thus SR and carbon sequestration. Nevertheless, few site studies have focused on the effects of water organic pollutants and eutrophication, which is a result of there being an excess of nutrients in water including nitrogen and phosphorus, on SR and SOC reservation ability of estuarine wetlands, as well as the relationship between pollutant interception and carbon sequestration of estuarine wetlands. Clarifying the effect of water pollution on SR and thus carbon sequestration, as well as the relationship between pollutant purification and carbon sequestration of estuarine wetlands will provide new understanding of estuarine wetland function and value.

The Yangtze River Delta, which is the most economically developed area of China, is now under great environmental pressure. The Jiuduansha wetland is the youngest original tidal wetland in the Yangtze River estuary and an important natural reserve in Shanghai with many important ecological functions [22]. This wetland connects the Yangtze River and the East China Sea and spans about 40 km from west to east. Similar to other estuarine areas, the Jiuduansha Wetland is also subject to organic pollutants and eutrophication. [22, 23]

In this study, two sampling zones in Jiudunsha Wetland, an upstream area (Shang shoal) and a downstream area (Xia shoal) were investigated. Specifically: 1) the variability in organic pollution and eutrophication between two shoals and its effects on SR of the estuarine wetland; 2) the microbial ecological mechanism through which these effects occurred; and 3) the relationship between pollution intercepting function and soil carbon sequestration of estuarine wetlands.

## Materials and Methods

### Ethics Statement

Shanghai Jiuduansha wetland Nature Reserve Administration granted permission to collect soil samples. We did not collect any endangered species. The GPS coordinates of the sample location are provided in the Materials and Methods section.

### Site Description

**Fig 1:**
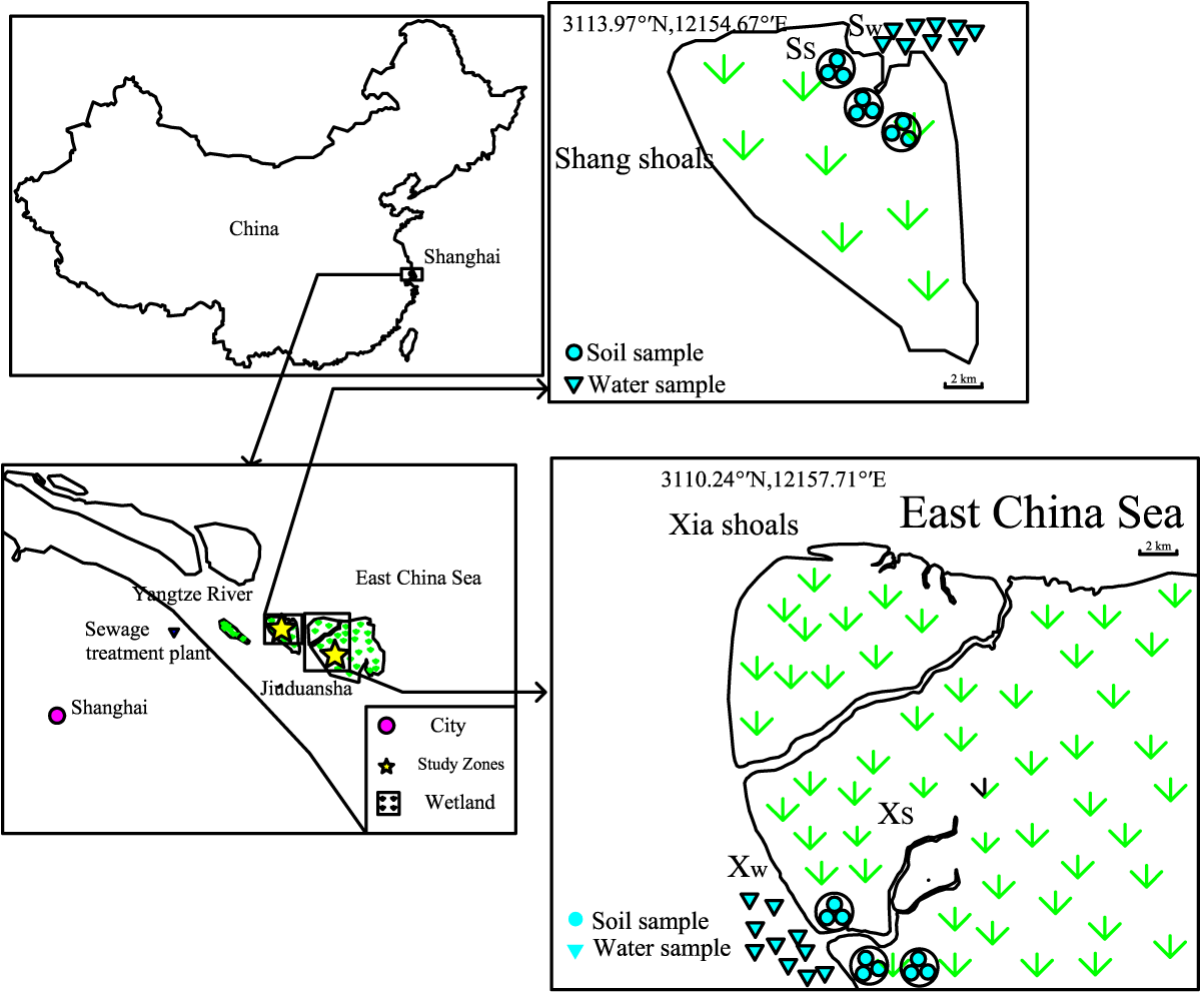
Map of the study areas of the Yangtze River estuary.

The Jiuduansha Wetland is located between the southern and northern watercourse of the Yangtze Estuary (31°03′–31°17′N, 121°46′–122°15′E), 12 km east of the Pudong International Airport (Fig 1). The wetland covers 423.2 km^2^ and consists of three shoals, Shang Shoal, Zhong Shoal and Xia Shoal. There is a large sewage treatment plant with effluent exit locating on upstream of Shang shoal. The Jiuduansha Wetland was designated as a National Wetland Nature Reserve in 2005. It is affected by the East Asian subtropical monsoon climate, has an average annual temperature of 17.3ºC, average summer temperature of 28.9ºC, and average winter temperature of 5.6ºC.

To highlight the influence of water pollution and eutrophication on soil respiration, two soil sampling zones with similar vegetation and soil structure [20], but that may have differing water quality, were set in Shang shoal and Xia Shoal (denoted S and X, respectively) (Table 1). Water flow from upstream is intercepted by the Shang Shoal before reaching Xia Shoal.

Three sites were set about 200 m apart from each other along the contour of each soil sampling zone (Shan shoal: S1, S2, S3; Xia shoal: X1, X2, X3), Each site consisted of three points approximately 20m apart (Shang shoals denoted Ss1 to Ss9; Xia shoals denoted Xs1 to Xs9). Nine random water points (Shang shoals denoted Sw1 to Sw9; Xia shoals denoted Xw1 to Xw9) were set in each water sampling zone near soil of shoals (about 100 m apart from each other). The details describing each sampling point are shown in Fig 1 and Table 1.

**Table 1:** The sampling zones station and basic hydrographic and vegetation properties^a^.

	Location	Average waterlogging time^b^(h/d)	Height(m)	Vegetation type	Vegetation biomass^d^(g/m^2^)
S^c^	3113.97,121 54.67	6.24	3.0~3.1	S. Mariqueter	492.85
X^c^	3110.25, 121 57.71	8.57	2.9~3.0	S. Mariqueter	571.63

^a^ The elevation data provided by East China Normal University estuary.

^b^ Tidal waterlogging time calculated according to the data in the tide table.

^c^ S: Shang shoals, X: Xia shoals.

^d^ Vegetation biomass data provided by State Key Laboratory of Pollution Control and Resources Reuse, College of Environmental Science and Engineering, Tongji University.

### Sampling and Pretreatment

Nine soil samples were collected from each sampling zone during January, April and July of 2011 and January of 2012. All samples were collected using the quincunx sampling method (1 m×1m), checked to ensure that they were free of major debris, and then packed in individual sterile plastic bags and immediately stored at 4°C. A portion of the fresh soil was processed immediately for SMR, soil microbial biomass (SMB), soil moisture and soil enzyme activity (sieved < 2 mm) analysis, while the rest was air-dried and stored at room temperature until assay of the other soil characteristics. All results reported are the means of triplicate analyses and expressed on an oven-dry basis. All sample analyses were conducted within 2 weeks. The remainder of the soil was stored at -70°C for subsequent DNA extraction.

A total of nine water samples were collected during each season in October 2010 and January, April and July 2011 for each zone. All water samples were collected from the surface water layer (-1 m to -3 m) using a 1000 ml single channel stainless steel sampler (FSR, Suzhou). Water was collected in 250 ml bottles and stored at 4°C until analysis, which was conducted as soon as possible.

## Analysis Methods

### SR and SMR

SR was measured using an automated soil respiration system (LI-8100, LI-COR, USA). Briefly, 10-cm-diameter polyvinyl chloride (PVC) soil collars were installed in each sampling point and SR was measured two times a day (day and night) at each point in each sampling season.

CO_2_ decomposed and released by microorganisms from 40 g original fresh soil samples incubated in 250 mL serum bottles within 24 h at 28°C was measured using a gas chromatograph (GC-14B, Shimadzu) with a stainless steel column (10 m × 2 mm) and a TCD detector [24, 25]. The column temperature, inlet temperature and detector temperature were 40°C, 40°C and 90°C, respectively. Nitrogen gas was applied as the carrier at a flow rate of 30 mL/min. The CO_2_ injection volume was 0.2 mL and the CO_2_ released per unit of time from microorganisms that were in the period between the adaptation phase and the logarithmic growth phase was assayed and reported as the SMR.

### Methodology and analysis of gene library construction

Microbial community of two soil samples (Shang shoal and Xia shoal), each which uniformly mixed with nine points (each point took samples at every season) were analyzed by gene library construction. Total DNA extraction was conducted using a FastDNA: emoji: spin kit for soil (Qbiogene Inc., USA). Extracted DNA was visualized on 1% agarose gels, after which it was stored at −20°C until subsequent analysis. To construct clone libraries of soil bacteria in study zones, PCR amplification of bacterial 16S rRNA gene fragments was performed using the universal bacterial primers 27f and 1492r [26]. PCR products were purified with a PCR Purification Kit (Biomiga Inc., USA). Briefly, 10 μl of the PCR products were cloned using the PMD18-T plasmid vector system (TaKaRa, Japan). Next, 5 μl of the ligation products were transformed into Escherichia coli JM109, which allows blue-white screening on LB plates containing ampicillin at 100 mg/mL, X-Gal at 20 mg/mL and IPTG at 40 mmol/L. Approximately 20% of positive clones were randomly selected for amplification by PCR with vector-specific, M13 primers and sequenced by a commercial service (BGI, China). The sequences were then compared with 16S rRNA genes in GenBank using the Basic Local Alignment Search Tool (BLAST) function. All consensus sequences were checked for chimeras using the CHIMERA CHECK program of the Ribosomal Database Project II and none were detected.

### Routine analysis

The SMB was estimated based on the Adenosine Triphosphate (ATP) levels [27], which were measured using an improved bioluminescent method as previously described [28].

The soil dehydrogenase and β-glucosidase activities were determined based on the standard method described by the Soil Science Society of America [29]. Above-ground plant tissues were oven dried at 80°C to a constant weight. The plant biomass reported is the sum of the above- and below-ground tissue per unit area (kg/m^2^). Other soil variables including the pH, soil moisture, salinity, soil organic carbon (SOC), ammonia nitrogen (NH_3_-N), nitrate nitrogen (NO_3_-N) and available phosphorus (AP) were assayed by routine methods [30, 31]. Water variables including the NH_3_-N, NO_3_-N, total phosphorus (TP) and total organic carbon (TOC) were assayed by routine methods [30].

### Statistical analysis

One-way ANOVA and Duncan’s multiple-comparison tests were conducted using the SPSS software (version 19.0, IBM SPSS Inc.) to determine the significance of the difference in soil microbial and abiotic properties between the two zones in each area. The results of each sampling point are the means of triplicate analyses. The results reported for each study zone are the means of the sampling points in the zone over a year period expressed on an oven-dry basis. Errors were reported as the standard deviation (SD) of the mean of nine sampling points in each study zone. Path analysis, which can determine the relationship among variables and give weight to a possible causal variable, was performed using the SPSS software (version 19.0, IBM SPSS Inc.). In addition, the correlation coefficient can be divided into direct and indirect effects, suggesting the relative importance of factors in a result.

## Results and Discussion

### Variability in water quality and soil physical and chemical characteristics between Shang and Xia shoals

As shown in Table 2, the average NH_3_-N, NO_3_-N and TP and organic pollutant levels differ in water in Shang and Xia shoal. TP, NH_3_-N and NO_3_-N and organic pollutants in Shang shoal water are higher than that of Xia shoal, which indicates greater eutrophication in Shang shoal than Xia shoal. A large sewage treatment plant is located upstream of Shang shoal, which may lead to higher organic pollutants and N/P in Shang shoal water than Xia shoal. The soil content of AP, NH_3_-N and NO_3_-N in the downstream Xia shoal was at a lower level than that in Shang shoal (see Table 3), which may have been due to the absorption function and blocking effect of Shang shoal on nutritive salts.

**Table 2:** Content of nitrogen and phosphorus and organic carbon in tidal waters of two sampling zones^a^.

	NH_3_-N(mg/L)^c^	NO_3_-N(mg/L) ^c^	TP(mg/L) ^c^	TOC(mg/L) ^c^
water in S^b^	2.54±1.40a^d^	9.58±1.01A	1.24±0.51A	17.16±8.91A
Water in X^b^	1.12±0.19b	8.78±1.73A	0.53±0.12B	8.37±3.65B

^a^ Seasonal average value

^b^ S: Shang shoals of Jiuduansha, X: Xia shoals of Jiuduansha.

^c^ TOC: total organic carbon. NH3-N: ammonia nitrogen.NO3-N: nitrate nitrogen. TP: total phosphorous.

^d^ The results reported for each study zone are the means of the sampling points in the zone. Errors were reported as the standard deviation (SD) of the mean 9 points in each study zone. Different capital letters means the significant difference between S and X at 0.01 level, different lower-case letters means the significant difference between X and S at 0.05 level.

**Table 3:** Basic physical and chemical properties of the sampling zones^a^.

	Soil pH	Salinity(g/kg)	Soil moisture	NH_3_-N^c^(mg/kg)	AP^c^ (mg/kg)	SOC^c^ (g/kg)
S^b^	7.29±0.15a^d^	2.90±0.12a	0.30±0.01a	15.06±1.36A	28.09±1.17A	3.99±0.66a
X^b^	7.33±0.29a	3.51±0.17b	0.29±0.01a	7.44±1.14B	12.21±4.53B	4.04±1.05a

^a^ Seasonal average value

^b^ S: Shang shoals of Jiuduansha, X: Xia shoals of Jiuduansha.

^c^ SOC: soil organic carbon. NH_3_-N: ammonia nitrogen. AP: available phosphorous.

^d^ Different capital letters means the significant difference between S and X at 0.01 level, different lower-case letters means the significant difference between X and S at 0.05 level. Errors were reported as the standard deviation (SD) of the mean of 9 points in each study zone.

The selected area had similar plant coverage and species (S1 Table); therefore, there were no significant differences in organic carbon input due to plant litter. The TOC in Shang shoal water was significantly higher than that in Xia shoal, indicating that there should be more organic matter as the tide enters soil in Shang shoal. Based on these findings, the SOC was expected to be higher in Shang shoal than in the Xia shoal; however, the opposite was true (see Table 3). These results suggest that the carbon output of Shang shoal soil through SR may be higher than that of Xia shoal.

To clarify the correlation between pollutant interception and SR of wetlands, the differences in SR and soil microbial activity between Shang shoal and Xia shoal were analyzed as follows.

### Variability in SR and soil microbial activity between Shang and Xia shoals and its relationship to soil characteristics and water quality

**Fig 2:**
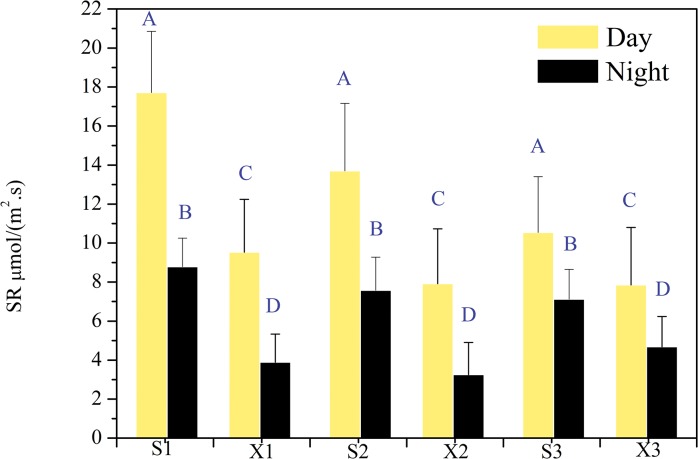
Seasonal average sites value of Soil respiration (SR) in Shang shoal and Xia shoal. S1~S3: three samples in Shang shoal, X1~X2: three samples in Xia shoal. Different capital letters (A, B, C, D) above the error bar represent significant difference between two areas at the 0.01 level.

S1 Table of the Supporting Information (SI) showed the average value of SR in three points from each site from spring to winter at day and night. It indicated that the values of SR from three sites at Shang shoal were higher than those of Xia shoal. Fig 2 shows the average SR of the four seasons at each site in Shang shoal and Xia shoal (both day and night).Overall, the SR intensity of each site in Shang shoal was significantly higher than that in Xia shoal during both day and night. (Average SR of day in Shang shoal is 65.8% higher than that in Xia shoal, average SR of night in Shang shoal is 98.3% higher than that in Xia shoal.)

**Fig 3:**
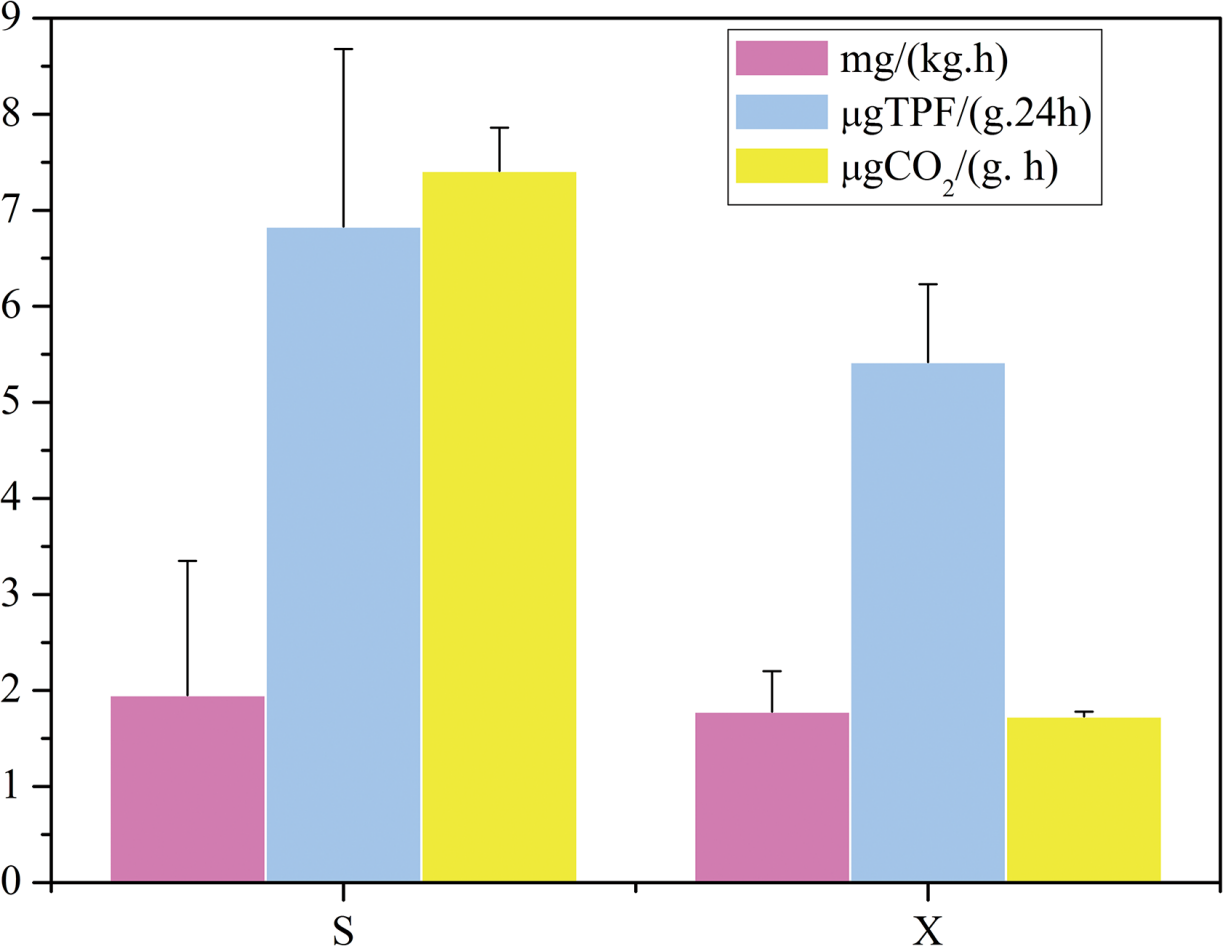
Soil microbial respiration (SMR), Dehydrogenase (DHA) and glycosidase (Gly) in Shang shoal and Xia shoal. S1~S3: three samples in Shang shoal, X1~X3: three samples in Xia shoal. Different capital letters (A, B) above the error bar represent significant difference between two areas at the 0.01 level.*The values were averaged by all sites in four seasons in each zone.

The average SMR and activities of soil enzymes associated with the metabolism of carbon (glycosidase and Dehydrogenase) were also higher in Shang shoal (Fig 3). Taken together, these results indicate that higher soil microbial activity in Shang shoal may lead to higher SR and therefore poor soil organic carbon sequestration capability and low SOC.

As shown in Table 4, Pearson correlation analysis revealed a significantly positive correlation between soil AP, NH_3_-N and SMR (P<0.01). These findings implied that the increased adsorption of TP, NH_3_-N and NO_3_-N in water by Shang shoal resulted in higher levels of NH_3_-N and AP in its soil, leading to higher soil enzyme activities and SMR, thus SR.

**Table 4:** Path analysis between SMR^a^ and basic physical and chemical properties.

		Indirect path coefficient						
Soil physic-chemical properties^b^	Pearson correlation	pH	Salinity	Moisture	NH_3_-N	AP	SOC	Total correlation
pH	0.003	1.000	-0.339	-0.318	0.369	0.218	-0.093	0.350
Salinity	-0.229	-0.339	1.000	-0.0837	-0.808	-0.824	0.466	-0.899
Moisture	-0.02	-0.318	0.111	1.000	-0.053	0.162	-0.389	-0.024
**NH**_**3**_ **-N**	0.588	0.369	-0.808	-0.053	1.000	0.849	-0.305	**0.975**
**AP**	0.239	0.218	-0.824	0.162	0.849	1.000	-0.377	**0.924**
SOC	0.006	-0.093	0.466	-0.389	-0.305	-0.377	1.000	-0.369

^a^Data of SMR in average value from Fig 3.

^b^Data of pH, salinity, moisture, NH_3_-N, AP and SOC from Table 3.

### Variability in soil microbial biomass and community structure between Shang and Xia Shoals

The differences in soil heterotrophic microbial activity may relate to microbial biomass and community structure, which is induced by differences in physical and chemical properties of soil.

SMB, which is represented by ATP concentration, is one of the main factors affecting microbial activity, especially the enzyme activity. The results showed that SMB in Shang shoal is higher than in Xia shoal (Shang shoal: (2.25±0.35A) E^-10^ATP mol/g (dry soil) Xia shoal: (9.57±2.87B) E^-11^ mol/g (dry soil)). In addition to microbial biomass, differences in community structure and dominant bacteria also led to variations in microbial activity.

The structure of soil microflora was determined from about 100 randomly selected clones from bacterial 16S rDNA libraries of each soil from Shang and Xia shoal (See S2 and S3 Tables). The two clone libraries shared ten phylogenetic groups, α-, β-, γ-, δ-, and ε-Proteobacteria, Acidobacteria, Bacteroidetes, Nitrospirae, Spirochaetes and Chloroflexi.

**Fig 4:**
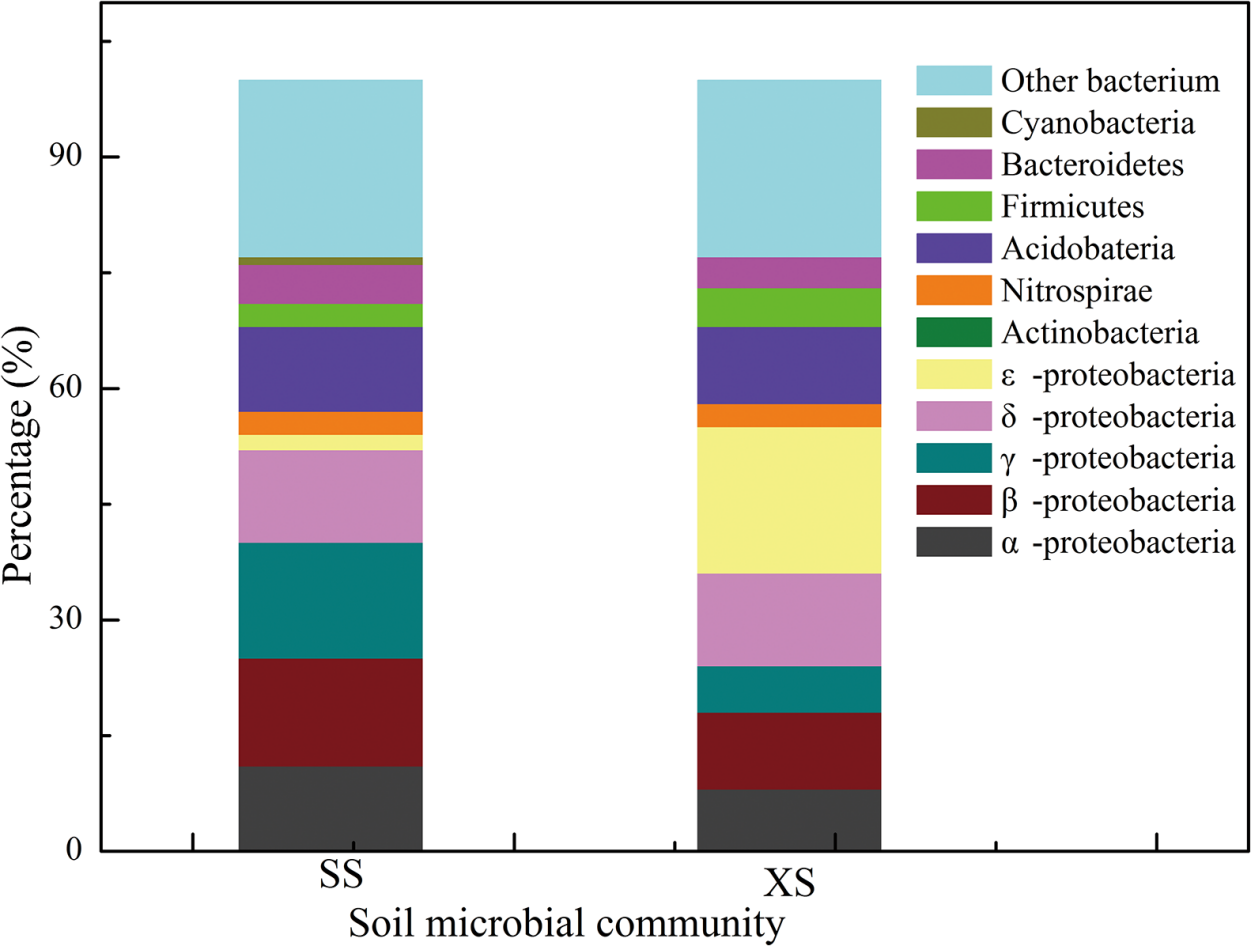
Prevalence of different phylogenetic groups of bacteria in Shang shoals and Xia shoals. S: Shang shoal, X: Xia shoal. Based on the data from S2 and S3 Tables, see SI.

The dominant bacteria in Shang and Xia shoals were Proteobacteria, which accounted for 54% and 55% of the total, respectively (Fig 4). Shang shoal soil primarily harbored γ-Proteobacteria (15%) and β-Proteobacteria (14%), while ε-Proteobacteria accounted for a smaller portion (2%, Fig 4). In Xia shoal soil, ε-Proteobacteria were dominant (19%), while β -Proteobacteria only accounted for 10% and α-Proteobacteria accounted for 8% (Fig 4), while δ-Proteobacteriain in the same proportion of Shang shoal and Xia shoal accounted for 12% (Fig 4).

In comparison, the content of γ -Proteobacteria and β-Proteobacteria in Shang shoal was greater than in Xia shoal, while ε-Proteobacteria was less abundant than in Xia shoal. Significant variation in structure and the dominant bacteria may also be an important cause of differences in microbial activity.

Overall, more serious organic pollution and eutrophication was observed in water around Shang shoal, which caused more absorption and interception of the NH_3_-N and AP by soil in Shang shoal soil, resulting in higher concentrations of NH_3_-N and AP relative to soil in Xia shoal. This might result in significant differences in microbial biomass, community structure and dominant bacteria when compared with Xia shoal soil, leading to differences in soil microbial activity and SMR, and finally affecting SR and soil carbon sequestration.

### Mechanism through which water organic pollution and eutrophication affect SR and thus soil carbon sequestration of estuarine wetlands

Nitrogen and phosphorus in water can be absorbed by soil. Numerous studies have shown that wetlands can absorb nitrogen and phosphorus in water. In fact, constructed wetlands comprise a cost-effective and efficient treatment technology for nitrogen removal from waste through soil adsorption and plant absorption that has been used increasingly in recent years [32]. The removal of NH_3_-N, NO_3_-N, TP and organic pollutants in water causes high soil NH_3_-N and AP content in wetlands because of soil interception and absorption.

In this study, the significantly higher soil NH_3_-N and AP of Shang shoal relative to Xia shoal may have been due to the increased absorption of N and P from Shang shoal water.

Many studies have shown that nitrogen and phosphorus and organic carbon input promoted the growth and metabolism of soil microbes, especially of some highly heterotrophic microbes [21, 33]. Both Antje and Wang found that supply and availability of organic carbon altered the distribution of microbial biomass and enzyme activities [34, 35]. Liu et al. found that, in a tropical forest, the addition of N and P will have interactive effects on soil microbes. In addition to directly promoting microbial metabolism and activity, N and P will indirectly affect microbial activity through its effects on microbial structure [36]. Wakelin et al. found that N has significant and long-term impacts on the size and structure of the soil microbial community at phylogenetic and functional levels and that amoA gene numbers were increased by N addition [37]. Zhang et al. reported that total nitrogen may have affected microbial community composition [11], especially the dominant bacterial species, and hence SMR. Barlett et al. demonstrated that β-Proteobacteria responded more to the addition of ammonium than nitrate. β-Proteobacteria also showed their peak relative abundance at the highest N: P ratio [38]. Studies of microbial activity and community structure in the Ljublja Marsh by Barbara Kraigher et al. suggested that some β-Proteobacteria and Acidobacteria were highly heterotopic [39]. Tang et al. also indicated that β- and δ-Proteobacteria have highly heterotrophic metabolism capability. However, on the other hand, some research report that ε- Proteobacteria is able to synthesize organic fixing CO_2_ [40]. Lutz-Arend et al found that with increasing eutrophication, the ratio of autotrophic to heterotrophic microbial processes becomes greatly reduced [41]. Zhang et al. showed that total organic carbon influenced soil microbial characteristics, which verifies the results of the present study [42].

The nitrogen and phosphorus content of tidal wetland soils is lower than that of farmland without fertilization, so its microbial activity is low. However, serious water organic pollution and nutrients from nearby wetlands will inevitably lead to greater absorption of nitrogen and phosphorus and organic matter in soil of wetlands, which may change the properties of soil and improve soil microbial activity and SR; thus, it is not conducive to soil carbon sequestration.

In the present study, soil in Shang shoal absorbed and intercepted eutrophic water, leading to higher NH_3_-N and AP content in the soil than in Xia shoal. Additionally, Pearson correlation analysis revealed a positive correlation between NH_3_-N, AP and SMR (P<0.01). This was likely because NH_3_-N and AP are conductive to the growth and reproduction of soil microorganisms, especially β- Proteobacteria, γ-Proteobacteria and Acidobacteria, while are unconducive to the growth of ε-Proteobacteria, which may affect SMR and SR. These findings are in accordance with the results of studies conducted by Wakelin et al. and Barlett et al. [37, 38].

In summary, increased water organic pollution and eutrophication near Shang shoal caused more absorption and interception of N, P and organic matter in Shang shoal soil, which led to reproduction and growth of highly heterotrophic metabolic microorganisms (such as β-,γ-Proteobacteria, Acidobacteria), which promoted soil microbial activity and SR and is not conducive to carbon sequestration when compared with Xia shoal. Conversely, the higher level of ε- Proteobacteria in Xia shoal, some of which is able to fix CO_2_ [40], may be another reason of its low SR.

### Correlation between soil organic carbon sequestration and pollutant purification of estuarine wetlands

Wetland plants fix large amounts of CO_2_ as organic carbon, but because of the unique characteristics of the wetland environment such as low temperature and long-term flooding, the soil heterotrophic microbial activity is weak, as is its ability to decompose organic carbon into CO_2_. Additionally, more plant organic carbon is retained in the soil of wetlands, which results in carbon sequestration [12, 43]. Pollutant purification is another important environmental function of wetlands [44]. In constructed wetlands, treatment performance has been accomplished through an integrated combination of biological, physical and chemical interactions among wetland components [45, 46]. As shown in Tables 2 and 3, pollution input of Shang shoal from water is higher than Xia shoal, which indicates the pollution intercepting function of wetlands. However, there is no significant difference on SOC between Shang shoal and Xia shoal, the SR of Shang shoal is higher than Xia shoal. These findings indicate that the degradation of input organic matters into CO_2_ and methane. In general, increased organic matter input results in increased microbial activity, which results in stronger SR and increased release of CO_2_ [47]. At the same time, greater interception and absorption of N and P will also increase the wetland soil microbial activity and SR, thus increasing organic matter degradation and CO_2_ output [30].

However, carbon in the organic pollutant input into wetlands from nearby water may not be directly from CO_2_; thus, the total CO_2_ input into wetland systems did not increase, while the release of CO_2_ increased due to increased SR resulted from more organic carbon input, thus wetlands may be transformed from carbon sinks to carbon sources.

As a result, increased water organic pollution and eutrophication near the wetland has probably resulted in its absorbing more N, P and organic matter, which likely enhanced SR and stimulated CO_2_ release, weakening the wetland carbon sequestration capacity.

Microbial heterotrophic activity of inland alpine wetlands is weak because of low temperature and long-term flooding; however, there are generally few people in areas around these systems so there are less exogenously input organic compounds and nitrogen/phosphorus, which may also be one of the reasons for its weak microbial activity. Due to the low microbial activity, organic carbon in plants, which originates from CO_2_, is not completely decomposed into CO_2_. This results in net carbon sequestrations of inland alpine wetlands, even though the organic carbon input from plants may be lower than that of estuary wetlands. Estuarine areas face more severe environmental stress and intercept more N, P and organic pollution. Although the quality of downstream water may be better after pollution purification by upstream estuarine wetlands, this may occur at the cost of reduced carbon sequestration function of upstream estuarine wetlands.

Reducing water pollution will reduce the N, P and TOC input to wetlands, resulting in reduced CO_2_ release of estuarine wetland soil.

In addition to a short development history and relatively high temperature, pollutant interception and purification may also explain why the soil carbon sequestration capability of estuarine wetlands is relatively low when compared to that of inland alpine wetlands, although its plant biomass may be higher than inland alpine wetland. To improve the carbon sequestration capability of estuarine wetlands, organic pollution and eutrophication of surrounding water systems needs to be effectively controlled and reduced. However, increasing the carbon sequestration capability of estuarine wetlands may occur at the cost of waste pollution purification function of estuarine wetlands. More general cases such as estuarine wetlands in the Yellow River and Pearl River estuaries currently being investigated to determine if these phenomena are widespread. Accordingly, future studies investigating methods of balancing the two environmental functions of estuarine wetlands are warranted.

## Conclusion

Based on the results of this study, the following conclusions were drawn: (1) N, P and organic pollutants in water of Shang shoal were higher than in Xia shoal, which caused increased absorption and interception of N, P and organic matter in Shang shoal soil. (2) Abundant N, P and organic carbon input (not from CO_2_) to soil of Shang shoal facilitated the reproduction and growth of highly heterotrophic metabolic microorganisms (such as β-, γ-Proteobacteria, Acidobacteria), which promoted soil microbial activity, and SR is not conducive to carbon sequestration in soil. (3) The estuary wetland is facing increasing pressure from environment pollution. The pollution purification function of estuarine wetlands may weaken their carbon sequestration function to some extent. Accordingly, it is necessary to continue to explore methods of balancing the two environmental functions of the estuary wetland.

## Supporting Information

S1 Table Seasonal values of Soil respiration (SR: μmol/(m^2^.s)) at day and night in Shang shoal and Xia shoal. S1-S3: three sites in Shang shoal of Jiuduansha, X1-X3: three sites in Xia shoal of Jiuduansha. Different capital letters means the significant difference between S and X at 0.01 level, errors were reported as the standard deviation (SD) of the mean of 3 points in each study site.
(DOCX)

S2 Table Clone library in Shang shoal. The structure of soil microflorawas determined from about 100 randomly selected clones from bacterial 16S rDNA libraries of soil from Shang shoal
(DOCX)

S3 Table Clone library in Xia shoal. The structure of soil microflorawas determined from about 100 randomly selected clones from bacterial 16S rDNA libraries of soil from Xia shoal
(DOCX)
