# Supplementary material for: Water Organic Pollution and Eutrophication Influence Soil Microbial Processes, Increasing Soil Respiration of Estuarine Wetlands: Site Study in Jiuduansha Wetland
Source: PLoS One. 2015 May 18;10(5):e0126951. doi: 10.1371/journal.pone.0126951 (PMC4436345; doi:10.1371/journal.pone.0126951)
Supplement: S1 Table — S1-S3: three sites in Shang shoal of Jiuduansha, X1-X3: three sites in Xia shoal of Jiuduansha. Different capital letters means the significant difference between S and X at 0.01 level, errors were reported as the standard deviation (SD) of the mean of 3 points in each study site. (DOCX) [file pone.0126951.s001.docx]

S1 Table. Seasonal values of Soil respiration (SR: μmol/(m^2^.s)) at day

and night in Shang shoal and Xia shoal

|  | | S1^a^ | S2 | S3 | X1^a^ | X2 | X3 |
| --- | --- | --- | --- | --- | --- | --- | --- |
| Spring | Day | 28.53±1.63A | 24.4±0.75A | 20.95±0.16A | 2.76±0.25B | 3.45±0.90B | 4.5±0.10B |
|  | Night | 11.77±1.16A | 9.57±0.20A | 14.54±0.06A | 2.51±0.06B | 2.68±0.36B | 4.26±0.98B |
| Summer | Day | 22.87±0.01A | 10.12±0.17A | 7.27±0.69A | 5.29±1.20B | 4.22±0.90B | 2.42±0.17B |
|  | Night | 14.54±0.01A | 9.40±0.18A | 7.10±0.17A | 4.81±0.30B | 4.21±0.50B | 3.62±0.02B |
| Autumn | Day | 14.06±0.73A | 16.34±0.16A | 12.37±0.58A | 3.33±0.03B | 5.40±0.18B | 3.97±0.43B |
|  | Night | 6.22±0.14A | 10.39±0.22A | 8.43±0.37A | 2.79±0.11B | 4.21±0.19B | 3.61±0.01B |
| Winter | Day | 5.40±0.28A | 3.95±0.01A | 1.58±0.09A | 1.18±0.38B | 0.78±0.18B | 0.57±0.30B |
|  | Night | 5.38±0.52A | 2.32±0.50A | 1.36±0.56A | 0.75±0.17B | 0.17±0.01B | 0.30±0.01B |

^a^ S1-S3: three sites in Shang shoal of Jiuduansha, X1-X3: three sites in Xia shoal of Jiuduansha

^b^Different capital letters means the significant difference between S and X at 0.01 level, errors were reported as the standard deviation (SD) of the mean of 3 points in each study site.
