# Supplementary material for: Water Organic Pollution and Eutrophication Influence Soil Microbial Processes, Increasing Soil Respiration of Estuarine Wetlands: Site Study in Jiuduansha Wetland
Source: PLoS One. 2015 May 18;10(5):e0126951. doi: 10.1371/journal.pone.0126951 (PMC4436345; doi:10.1371/journal.pone.0126951)
Supplement: S2 Table — The structure of soil microflorawas determined from about 100 randomly selected clones from bacterial 16S rDNA libraries of soil from Shang shoal (DOCX) [file pone.0126951.s002.docx]

S2 Table. Clone library in Shang shoal. (The structure of soil microflorawas determined from about 100 randomly selected clones from bacterial 16S rDNA libraries of soil from Shang shoal.)

| Description | NCBI Number | Duplicate | Similarity /% |
| --- | --- | --- | --- |
| α-proteobacteria -11 | | | |
| Hyphomonasjannaschiana strain DSM 5153 16S ribosomal RNA gene, partial sequence | KF863146.1 | 1 | 99 |
| Porphyrobacterdokdonensis strain DSW-74 16S ribosomal RNA gene, partial sequence | NR_043469.1 | 2 | 99 |
| Uncultured Alphaproteobacteria bacterium 16S rRNA gene from clone QEDN5AG01 | CU926364.1 | 1 | 100 |
| Uncultured Alphaproteobacteria bacterium 16S rRNA gene from clone QEDS2DF09 | CU921643.1 | 1 | 100 |
| Labrenzia sp. MBE 8 16S ribosomal RNA gene, partial sequence | KF724485.1 | 1 | 100 |
| Uncultured alpha proteobacterium clone C-5-25 16S ribosomal RNA gene, partial sequence | KF827099.1 | 1 | 99 |
| Uncultured alpha proteobacterium clone IC3100 16S ribosomal RNA gene, partial sequence | HQ595222.1 | 1 | 96 |
| Uncultured alpha proteobacterium clone C-5-25 16S ribosomal RNA gene, partial sequence | KF827099.1 | 1 | 99 |
| Uncultured Rickettsiales bacterium clone YL197 16S ribosomal RNA gene, partial sequence | HM856558.1 | 2 | 95 |
| β-proteobacteria-14 | | | |
| Azoarcus sp. KH32C DNA, complete genome | AP012304.1 | 2 | 97 |
| Uncultured beta proteobacterium clone JOT245-88(R23) 16S ribosomal RNA gene, partial sequence | HQ909308.1 | 1 | 98 |
| Uncultured beta proteobacterium clone 40_3_81 16S ribosomal RNA gene, partial sequence | JF413041.1 | 1 | 98 |
| Uncultured beta proteobacterium clone MPWIC_G01 16S ribosomal RNA gene, partial sequence | EF414170.1 | 2 | 97 |
| Uncultured beta proteobacterium partial 16S rRNA gene, clone O:RM-D7 | HE974835.1 | 2 | 98 |
| Uncultured beta proteobacterium clone MBMV10 16S ribosomal RNA gene, partial sequence | FJ538157.1 | 2 | 98 |
| Uncultured beta proteobacterium clone P-R112 16S ribosomal RNA gene, partial sequence | JN038891.1 | 1 | 97 |
| Uncultured beta proteobacterium clone TDNP_Bbc97_34_3_102 16S ribosomal RNA gene, partial sequence | FJ516802.1 | 1 | 99 |
| Uncultured beta proteobacterium clone TH_z8 16S ribosomal RNA gene, partial sequence | GU998911.1 | 1 | 99 |
| Uncultured beta proteobacterium clone II6C 16S ribosomal RNA gene, partial sequence | FJ205321.1 | 1 | 99 |
| γ-proteobacteria-15 | | | |
| Methylocystis sp. 50/54 partial 16S rRNA gene, strain 50/54 | AJ458510.1 | 1 | 92 |
| Uncultured gamma proteobacterium clone 5m-95 16S ribosomal RNA gene, partial sequence | GU061264.1 | 1 | 99 |
| Uncultured gamma proteobacterium partial 16S rRNA gene, isolate 2.1, clone 56b | HE804020.1 | 1 | 98 |
| Uncultured gamma proteobacterium clone ONGS225 16S ribosomal RNA gene, partial sequence | JX241017.1 | 1 | 97 |
| Uncultured gamma proteobacterium clone OTU30 16S ribosomal RNA gene, partial sequence | KF956499.1 | 1 | 99 |
| Uncultured gamma proteobacterium clone BS1-0-28 16S ribosomal RNA gene, partial sequence | AY254913.1 | 1 | 97 |
| Uncultured hydrocarbon seep bacterium BPC023 16S ribosomal RNA gene, partial sequence | AF154087.1 | 1 | 97 |
| Uncultured gamma proteobacterium clone 5m-95 16S ribosomal RNA gene, partial sequence | GU061264.1 | 1 | 99 |
| Uncultured gamma proteobacterium clone V1B07b156 16S ribosomal RNA gene, partial sequence | GU369933.1 | 1 | 96 |
| Uncultured gamma proteobacterium clone SS1_B_07_30 16S ribosomal RNA gene, partial sequence | EU050787.1 | 1 | 98 |
| Uncultured gamma proteobacterium partial 16S rRNA gene, clone P38 | HE648209.1 | 1 | 99 |
| Uncultured hydrocarbon seep bacterium BPC023 16S ribosomal RNA gene, partial sequence | AF154087.1 | 1 | 100 |
| Uncultured Methylosarcina sp. clone Xh_Meth1b_CA37 16S ribosomal RNA gene, partial sequence | JQ038198.1 | 1 | 97 |
| Uncultured gamma proteobacterium clone 5m-11 16S ribosomal RNA gene, partial sequence | GU061213.1 | 2 | 99 |
| ε-proteobacteria-2 | | | |
| Arcobacteranaerophilus partial 16S rRNA gene, strain JC83, isolate D09-60 | FR686495.1 | 1 | 94 |
| Uncultured epsilon proteobacterium partial 16S rRNA gene, isolate 1.2, clone 1b_13 | HE803939.1 | 1 | 99 |
| δ-proteobacteria-12 | | | |
| Uncultured Desulfobacterales bacterium clone SFeB33 16S ribosomal RNA gene, partial sequence | JQ723620.1 | 1 | 97 |
| Desulforhopalussingaporensis strain S'pore T1 16S ribosomal RNA gene, partial sequence | NR_028742.1 | 1 | 95 |
| Uncultured delta proteobacterium clone ARTE12_256 16S ribosomal RNA gene, partial sequence | GU230395.1 | 1 | 96 |
| Uncultured Geobacter sp. clone VHS-B3-70 16S ribosomal RNA gene, partial sequence | DQ394958.1 | 1 | 90 |
| Uncultured delta proteobacterium partial 16S rRNA gene, clone Sylt 33 | AM040129.1 | 1 | 98 |
| Uncultured delta proteobacterium clone MA-R61 16S ribosomal RNA gene, partial sequence | JN038629.1 | 1 | 99 |
| Delta proteobacterium enrichment culture clone MC18B6-22 16S ribosomal RNA gene, partial sequence | JQ256504.1 | 1 | 98 |
| Uncultured delta proteobacterium clone 118 0th day 16S ribosomal RNA gene, partial sequence | JQ860941.1 | 1 | 97 |
| Uncultured delta proteobacterium clone M-B211 16S ribosomal RNA gene, partial sequence | JN038932.1 | 2 | 98 |
| Uncultured delta proteobacterium clone YS-UMF1_C157 16S ribosomal RNA gene, partial sequence | DQ901586.1 | 1 | 98 |
| Uncultured delta proteobacterium clone TH_e46 16S ribosomal RNA gene, partial sequence | EU980141.1 | 1 | 97 |
| Nitrospirae-3 | | | |
| Uncultured Nitrospirae bacterium partial 16S rRNA gene, clone E80B0W9 | HE613585.1 | 1 | 98 |
| Uncultured Nitrospirae bacterium clone M1 16S ribosomal RNA gene, partial sequence | FJ178584.1 | 1 | 99 |
| Uncultured Nitrospirales bacterium gene for 16S rRNA, partial sequence, clone: IODP1324B6H3.80 | AB448916.1 | 1 | 96 |
| Acidobacteria-11 | | | |
| Uncultured Acidobacteria bacterium clone ANOX-122 16S ribosomal RNA gene, partial sequence | JF344684.1 | 2 | 96 |
| Uncultured Acidobacteriaceae bacterium partial 16S rRNA gene, clone Sylt 38 | AM040134.1 | 1 | 94 |
| Uncultured Acidobacteria bacterium clone Aug-PC328 16S ribosomal RNA gene, partial sequence | JQ795399.1 | 1 | 97 |
| Uncultured Acidobacteria bacterium clone Therm30-A11 16S ribosomal RNA gene, partial sequence | AY533887.1 | 1 | 96 |
| Uncultured Acidobacterium sp. clone HCM3MC83_2C_FL 16S ribosomal RNA gene, partial sequence | EU373967.1 | 2 | 96 |
| Uncultured Acidobacteria bacterium clone P-B284 16S ribosomal RNA gene, partial sequence | JN039004.1 | 1 | 99 |
| Uncultured bacterium clone 1H3M_18 16S ribosomal RNA gene, partial sequence | JN230074.1 | 1 | 99 |
| Uncultured Acidobacteria bacterium clone G23 16S ribosomal RNA gene, partial sequence | HQ162726.1 | 2 | 99 |
| Firmicutes-3 | | | |
| Uncultured Firmicutes bacterium gene for 16S rRNA, partial sequence, isolate: sd-jx73 | AB690763.1 | 1 | 99 |
| Uncultured Clostridiales bacterium partial 16S rRNA gene, clone II-A-G3 | FR774779.1 | 2 | 97 |
| Bacteroidetes-5 | | | |
| Uncultured Bacteroidetes bacterium clone GASP-WC2S1_H10 16S ribosomal RNA gene, partial sequence | EF074888.1 | 1 | 95 |
| Uncultured Bacteroidetes bacterium clone OTU-45 16S ribosomal RNA gene, partial sequence | JQ794632.1 | 2 | 97 |
| uncultured Bacteroidetes bacterium partial 16S rRNA gene, isolate 1.2, clone 174 | HE803905.1 | 1 | 97 |
| Uncultured Flavobacterium sp. clone SV-5 16S ribosomal RNA gene, partial sequence | GU233829.1 | 1 | 99 |
| Cyanobacteria -1 | | | |
| Uncultured Cyanobacterium sp. clone CM01188X1G03 16S ribosomal RNA gene, partial sequence | GU170789.1 | 1 | 99 |
| Others-23 | | | |
| Uncultured Chloroflexi bacterium clone TRF-183 16S ribosomal RNA gene, partial sequence | JX859926.1 | 1 | 96 |
| Uncultured bacterium clone aab67g10 16S ribosomal RNA gene, partial sequence | DQ814382.1 | 1 | 99 |
| Uncultured Verrucomicrobia bacterium clone LD1-PB20 16S ribosomal RNA gene, partial sequence | AY114334.1 | 1 | 98 |
| Uncultured Verrucomicrobia bacterium clone LD1-PB20 16S ribosomal RNA gene, partial sequence | AY114334.1 | 1 | 98 |
| Uncultured Verrucomicrobia bacterium clone SGR80 16S ribosomal RNA gene, partial sequence | JQ793450.1 | 1 | 96 |
| uncultured bacterium clone TopBa24 16S ribosomal RNA gene, partial sequence | EF999354.1 | 1 | 98 |
| Uncultured bacterium clone w3uc25 16S ribosomal RNA gene, partial sequence | DQ416527.1 | 1 | 96 |
| Uncultured bacterium clone C8S-75 16S ribosomal RNA gene, partial sequence | EU652582.1 | 2 | 98 |
| Uncultured bacterium clone sa23.18 16S ribosomal RNA gene, partial sequence | HQ904157.1 | 1 | 99 |
| Uncultured bacterium clone B-55 16S ribosomal RNA gene, partial sequence | HQ703856.1 | 1 | 99 |
| Uncultured bacterium clone 060329_T2S4_S_T_SDP_140 small subunit ribosomal RNA gene, partial sequence | FJ351928.1 | 1 | 100 |
| Sideroxydanslithotrophicus strain ES-1 16S ribosomal RNA gene, complete sequence | NR_074731.1 | 1 | 97 |
| Uncultured bacterium clone 3051bac1-86 16S ribosomal RNA gene, partial sequence | GU982773.1 | 1 | 92 |
| Uncultured bacterium clone 16S_JLJ4_73 16S ribosomal RNA gene, partial sequence | KC925446.1 | 1 | 96 |
| Uncultured bacterium clone BCSAS2P1D6 16S ribosomal RNA gene, partial sequence | FJ665204.1 | 2 | 95 |
| Uncultured bacterium gene for 16S ribosomal RNA, partial sequence, clone: YU-21-F | AB780324.1 | 1 | 98 |
| Uncultured bacterium clone VC66 16S ribosomal RNA gene, partial sequence | EU593790.1 | 1 | 99 |
| Uncultured bacterium gene for 16S rRNA, partial sequence, clone: Hados.Water.Eubac.6 | AB355047.1 | 1 | 99 |
| Unidentified marine bacterioplankton clone P2-1-1B_69 16S ribosomal RNA gene, partial sequence | KC000820.1 | 1 | 96 |
| Uncultured Gemmatimonadetes bacterium clone 4_59 16S ribosomal RNA gene, partial sequence | KC009992.1 | 1 | 99 |
| Uncultured bacterium gene for 16S ribosomal RNA, partial sequence, clone: 87-061020-Higata-7 | AB560012.1 | 1 | 99 |
| Total | | 100 |  |
