# Supplementary material for: Water Organic Pollution and Eutrophication Influence Soil Microbial Processes, Increasing Soil Respiration of Estuarine Wetlands: Site Study in Jiuduansha Wetland
Source: PLoS One. 2015 May 18;10(5):e0126951. doi: 10.1371/journal.pone.0126951 (PMC4436345; doi:10.1371/journal.pone.0126951)
Supplement: S3 Table — The structure of soil microflorawas determined from about 100 randomly selected clones from bacterial 16S rDNA libraries of soil from Xia shoal (DOCX) [file pone.0126951.s003.docx]

S3 Table. Clone library in Xia shoal.( The structure of soil microflorawas determined from about 100 randomly selected clones from bacterial 16S rDNA libraries of soil from Xia shoal.)

| Description | NCBI Number | Duplicate | Similarity /% |
| --- | --- | --- | --- |
| α-proteobacteria-8 | | | |
| Alpha proteobacterium LM-1 16S ribosomal RNA gene, partial sequence | JF490044.1 | 1 | 99 |
| Uncultured Alpha proteobacterium partial 16S rRNA gene, clone AMLD9 | AM934958.1 | 1 | 99 |
| Pleomorphomonasoryzae gene for 16S rRNA, partial sequence, strain: NBRC 102288 | AB681744.1 | 1 | 99 |
| Rhizobiales bacterium RR47 gene for 16S ribosomal RNA, partial sequence | AB174816.1 | 2 | 96 |
| Rhodospirillales bacterium L96 partial 16S rRNA gene, isolate L96 | AM913948.1 | 1 | 90 |
| Uncultured Agrobacterium sp. clone AV_8R-S-F05 16S ribosomal RNA gene, partial sequence | EU341285.1 | 1 | 98 |
| Uncultured Hyphomicrobiaceae bacterium clone AUVE_06B04 16S ribosomal RNA gene, partial sequence | EF651264.1 | 1 | 96 |
| β-proteobacteria-10 | | | |
| Beta proteobacterium HTCC349 16S ribosomal RNA gene, partial sequence | AY429717.1 | 1 | 98 |
| Uncultured beta proteobacterium gene for 16S rRNA, partial sequence, clone: Jy04A14 | AB591389.1 | 1 | 96 |
| Uncultured beta proteobacterium clone RBE2CI-57 16S ribosomal RNA gene, partial sequence | EF111164.1 | 1 | 99 |
| Uncultured beta proteobacterium clone MEf05cnp11C8 16S ribosomal RNA gene, partial sequence | FJ828028.1 | 1 | 98 |
| Uncultured beta proteobacterium clone MA-R6 16S ribosomal RNA gene, partial sequence | JN038589.1 | 1 | 95 |
| Uncultured beta proteobacterium gene for 16S rRNA, partial sequence, clone: UH-50 | AB265944.2 | 1 | 98 |
| Uncultured beta proteobacterium clone MA-R26 16S ribosomal RNA gene, partial sequence | JN038607.1 | 1 | 99 |
| Uncultured beta proteobacterium clone P-R54 16S ribosomal RNA gene, partial sequence | JN038841.1 | 1 | 99 |
| Hydrogenophaga sp. a9 16S ribosomal RNA gene, partial sequence | EF179863.1 | 1 | 99 |
| Uncultured Thiobacillus sp. partial 16S rRNA gene, clone P24 | HE648196.1 | 1 | 99 |
| γ-proteobacteria-6 | | | |
| Acinetobacter sp. CN1b(2011) 16S ribosomal RNA gene, partial sequence | JN228299.1 | 1 | 99 |
| Uncultured Methylosarcina sp. clone Xh_Meth1b_CA37 16S ribosomal RNA gene, partial sequence | JQ038198.1 | 3 | 99 |
| Tolumonasauensis DSM 9187, complete genome | CP001616.1 | 1 | 99 |
| Uncultured Xanthomonas sp. clone g47 16S ribosomal RNA gene, partial sequence | DQ167066.1 | 1 | 99 |
| δ-proteobacteria-12 | | | |
| Uncultured delta proteobacterium clone AS-45-11 16S ribosomal RNA gene, partial sequence | GQ406161.1 | 1 | 92 |
| Uncultured delta proteobacterium clone GC0AA5ZH07PP1 16S ribosomal RNA gene, partial sequence | JQ919704.1 | 1 | 97 |
| Uncultured delta proteobacterium clone ZLL-E89 16S ribosomal RNA gene, partial sequence | JF807043.1 | 2 | 98 |
| Uncultured Delta-proteobacteria bacterium 16S rRNA gene from clone QEDV1BD01 | CU919561.1 | 1 | 87 |
| Uncultured Anaeromyxobacter sp. gene for 16S rRNA, partial sequence, clone: LH-45 | AB265868.2 | 1 | 98 |
| Uncultured Anaeromyxobacter sp. gene for 16S rRNA, partial sequence, clone: UH-35 | AB265929.2 | 1 | 92 |
| Uncultured Geobacter sp. partial 16S rRNA gene, clone II-A-G40 | FR774793.1 | 1 | 96 |
| Uncultured Geobacter sp. clone 18-00_Iron-G1 16S ribosomal RNA gene, partial sequence | HM217296.1 | 1 | 98 |
| Desulfuromonadales bacterium JN18_A94_J 16S ribosomal RNA gene, partial sequence | DQ168651.1 | 1 | 97 |
| Uncultured Desulfobacca sp. clone G22 16S ribosomal RNA gene, partial sequence | HQ162725.1 | 1 | 99 |
| Uncultured Syntrophaceae bacterium clone Sy70_DA12 16S ribosomal RNA gene, partial sequence | GU472591.1 | 1 | 92 |
| ε-proteobacteria-19 | | | |
| Uncultured epsilon proteobacterium 1054 gene for 16S ribosomal RNA, partial sequence, clone: 1054 | AB030602.1 | 2 | 99 |
| Uncultured epsilon proteobacterium clone ATB-LH-6148 16S ribosomal RNA gene, partial sequence | FJ535178.1 | 1 | 92 |
| Uncultured epsilon proteobacterium clone CC1_cl15 16S ribosomal RNA gene, partial sequence | DQ295570.1 | 1 | 98 |
| Uncultured epsilon proteobacterium clone CC1_cl51 16S ribosomal RNA gene, partial sequence | DQ295579.1 | 1 | 99 |
| Uncultured epsilon proteobacterium clone G452 16S ribosomal RNA gene, partial sequence | HQ162754.1 | 9 | 100 |
| Uncultured epsilon proteobacterium clone THc2-43 16S ribosomal RNA gene, partial sequence | HM854353.1 | 2 | 99 |
| Uncultured Sulfurospirillum sp. gene for 16S ribosomal RNA, partial sequence, clone: AK25 | AB713999.1 | 1 | 99 |
| Sulfuricurvum sp. enrichment culture clone D2CL_Bac_16S_Clone8 16S ribosomal RNA gene, partial sequence | EU498374.1 | 1 | 99 |
| Sulfuricurvum sp. enrichment culture clone D2CL_Bac_16S_Clone8 16S ribosomal RNA gene, partial sequence | EU498374.1 | 1 | 97 |
| Nitrospirae-3 | | | |
| Uncultured Nitrospirae bacterium clone H06_WMSP2 16S ribosomal RNA gene, partial sequence | DQ450808.1 | 1 | 99 |
| Uncultured Nitrospira sp. gene for 16S rRNA, partial sequence, clone: Jy04A34 | AB591403.1 | 1 | 100 |
| Uncultured Nitrospirae bacterium clone M1 16S ribosomal RNA gene, partial sequence | FJ178584.1 | 1 | 100 |
| Acidobacteria10 | | | |
| Uncultured Acidobacteria bacterium clone B5A 16S ribosomal RNA gene, partial sequence | FJ205239.1 | 1 | 99 |
| Uncultured Acidobacteria bacterium clone G23 16S ribosomal RNA gene, partial sequence | HQ162726.1 | 1 | 99 |
| Uncultured Acidobacteria bacterium clone P-B284 16S ribosomal RNA gene, partial sequence | JN039004.1 | 2 | 99 |
| Uncultured Acidobacteria bacterium clone P-R75 16S ribosomal RNA gene, partial sequence | JN038862.1 | 2 | 98 |
| Uncultured Acidobacteria bacterium partial 16S rRNA gene, clone L326 | AM491121.1 | 1 | 97 |
| Uncultured Acidobacteria bacterium clone GASP-MB3W1_B10 16S ribosomal RNA gene, partial sequence | EF665818.1 | 1 | 95 |
| Uncultured Acidobacteria bacterium partial 16S rRNA gene, clone L326 | AM491121.1 | 1 | 97 |
| Uncultured Acidobacteria bacterium clone SEG_08_694 16S ribosomal RNA gene, partial sequence | HQ729900.1 | 1 | 98 |
| Firmicutes-5 | | | |
| Uncultured Firmicutes bacterium gene for 16S rRNA, partial sequence, clone: RL-B5 | AB742062.1 | 3 | 95 |
| Uncultured Firmicutes bacterium 16S rRNA gene from clone QEDN2BB09 | CU926241.1 | 2 | 99 |
| Bacteroidetes-4 | | | |
| Uncultured Bacteroidetes bacterium clone wn100 16S ribosomal RNA gene, partial sequence | JQ012322.1 | 2 | 92 |
| Uncultured Bacteroidetes bacterium clone MVS-54 16S ribosomal RNA gene, partial sequence | DQ676403.1 | 1 | 97 |
| Uncultured Bacteroidetes bacterium 16S rRNA gene from clone QEDP1BF06 | CU924663.1 | 1 | 93 |
| Others-23 | | | |
| Uncultured Chloroflexi bacterium clone Alchichica_AQ1_2_1B_115 small subunit ribosomal RNA gene, partial sequence | JN825473.1 | 1 | 90 |
| Uncultured Chloroflexi bacterium clone LQH290 16S ribosomal RNA gene, partial sequence | JN868187.1 | 1 | 99 |
| Uncultured eubacterium clone U12-3 16S ribosomal RNA gene, partial sequence | DQ137893.1 | 1 | 93 |
| Uncultured Syntrophomonas sp. clone wn73 16S ribosomal RNA gene, partial sequence | JQ012299.1 | 1 | 96 |
| Uncultured Gemmatimonadetesbacterium clone 4_59 16S ribosomal RNA gene, partial sequence | KC009992.1 | 1 | 99 |
| Uncultured Verrucomicrobiae bacterium clone HCM3MC80_2A_FF 16S ribosomal RNA gene, partial sequence | EU373983.1 | 1 | 91 |
| Uncultured Verrucomicrobia bacterium clone C294 16S ribosomal RNA gene, partial sequence | JF833738.1 | 1 | 95 |
| Uncultured Verrucomicrobia bacterium clone GASP-MA2W2_G06 16S ribosomal RNA gene, partial sequence | EF663260.1 | 1 | 97 |
| CandidatusHaliscomenobactercalcifugiens partial 16S rRNA gene, clone MS-uSonn1-G | AJ786328.1 | 1 | 92 |
| Uncultured Chlorobi bacterium clone Cart-N2 16S ribosomal RNA gene, partial sequence | AY118151.1 | 1 | 95 |
| Uncultured Cytophagales bacterium clone TDNP_USbc97_38_3_137 16S ribosomal RNA gene, partial sequence | FJ516965.1 | 3 | 96 |
| Uncultured Lutibacter sp. clone SFeB27 16S ribosomal RNA gene, partial sequence | JQ723619.1 |  | 99 |
| Uncultured rape rhizosphere bacterium wr0070 partial 16S rRNA gene | AJ295516.1 | 1 | 98 |
| Denitrifying bacterium enrichment culture clone NOA_1_D7 16S ribosomal RNA gene, partial sequence | FJ802175.1 | 2 | 99,96 |
| Denitrifying bacterium enrichment culture clone NOB_2_F9 16S ribosomal RNA gene, partial sequence | FJ802253.1 | 1 | 95 |
| Sideroxydanslithotrophicus ES-1, complete genome | CP001965.1 | 2 | 94,99 |
| Iron-reducing bacterium enrichment culture clone FEA_2_D7 16S ribosomal RNA gene, partial sequence | FJ802334.1 | 1 | 99 |
| Iron-reducing bacterium enrichment culture clone HN70 16S ribosomal RNA gene, partial sequence | FJ269065.1 | 1 | 90 |
| Uncultured bacterium partial 16s rRNA gene, clone D41S30C44 | FM956927.1 | 1 | 98 |
| Total | | 100 |  |
